# PCD Increases Content About Public Health Approaches Being Implemented to Improve Population Health

**DOI:** 10.5888/pcd15.180644

**Published:** 2018-12-20

**Authors:** Leonard Jack

**Figure:**
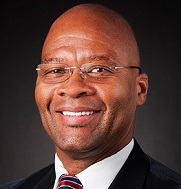
Leonard Jack Jr, PhD, MSc

*Preventing Chronic Disease* (PCD) is a valuable resource for researchers, evaluators, and policy makers. PCD receives submissions on various public health topics from authors all over the world. From January through mid-November 2018, PCD received over 600 new submissions and 140 resubmissions. It was a tremendously successful year during which PCD published rich, relevant, and timely content. In April, PCD published findings from the journal’s first-ever external review panel (1), which resulted in the following vision and mission statements:

**Vision.** PCD will serve as an influential journal in the dissemination of proven and promising public health findings, innovations, and practices with editorial content respected for its integrity and relevance to chronic disease prevention.
**Mission statement.** The mission of PCD is to promote dialogue among researchers, practitioners, and policy makers worldwide on the integration and application of research findings and practical experience to improve population health.

PCD used its new vision and mission statements to further refine priority content areas (1) and published high-quality articles that aligned with the following 4 priority areas:

- Development, implementation, and evaluation of population-based interventions to prevent chronic diseases and control their effect on quality of life, illness, and death.
- Behavioral, psychological, genetic, environmental, biological, and social factors that influence health.
- Interventions that reduce the disproportionate incidence of chronic diseases among at-risk populations.
- Development, implementation, and evaluation of public health law and health-policy–driven interventions.

## PCD Focuses on Results-Driven Publications

Most articles published this year across the journal’s 4 focus areas presented results — moving beyond merely describing theoretical frameworks, program descriptions, and partnerships and collaborations. PCD was intentional in communicating to its readership that submissions focusing on discussions on partnership formation, coalition building, program overviews, frameworks, or anecdotal lessons learned will not be given high priority. PCD’s priority in 2018 was to identify submissions that provided careful, thoughtful, and rigorously conducted research and evaluation findings derived from the application of population-based approaches to ameliorate the negative effects of chronic disease on population health.

Toward this end, PCD offered authors opportunities to report findings by using 2 new article types, Implementation Evaluation and Program Evaluation Briefs. From January to November, PCD received over 40 submissions of these article types. Implementation Evaluation articles provide insights into factors that affect the ability of public health practice to successfully package and disseminate effective interventions that have been implemented and evaluated in real-world settings. The Implementation Evaluation articles published this year addressed such topics as adopting obesity interventions in school districts and community programs, examining the cost-effectiveness of a nutrition and wellness program, and developing and implementing a surveillance system to monitor mental health. Program Evaluation Briefs were offered to encourage more submissions with findings from well-delivered and evaluated public health programs. Several program evaluation articles are scheduled for publication early in 2019.

## PCD Published Record Number of Collections

A major recommendation from the external review panel was to advance PCD efforts to identify articles reporting findings from researchers and practitioners working in various settings, using population-based interventions and policies to improve health outcomes. PCD collections (also referred to as supplements) consist of articles addressing pressing public health issues. PCD collections allow PCD readers to gain access to current research and evaluation findings, advances in methods and data collection, and recommendations to advance future research, evaluation, and programmatic activities.

### 2018 Collections

In January, PCD published its first collection of the year, “State and Local Public Health Actions to Prevent and Control Chronic Diseases” (2). This collection describes the coordinated approach to prevent and control chronic disease by the Centers for Disease Control and Prevention’s funded partners. Specifically, this collection discusses evaluation and programmatic approaches used by state and local health departments (with differing levels of capacity) to reduce the burden of chronic disease. The collection consists of 12 articles providing insights on a range of programmatic and health outcomes at the national, state, and local levels (https://www.cdc.gov/pcd/collections/pdf/PCD_StateAndLocal_Collection_FINAL_3-27-18.pdf).

PCD released its second collection of the year in February. This collection featured student paper winners in the journal’s 2017 Student Research Paper Contest (3). The 2017 student paper contest involved over 70 student manuscripts that underwent careful internal and external review. PCD identified 5 student paper winners — a winner each in the high school, undergraduate, and graduate categories and 2 winners in the doctoral category. PCD readers are encouraged to take a look at this impressive body of work generated by some of the nation’s brightest emerging scholars (https://www.cdc.gov/pcd/collections/pdf/PCD_2017StudentPapersCombined.pdf).

In March, PCD released a third collection, “The Childhood Obesity Research Demonstration (CORD) Project” (4). The CORD project implemented evidence-based interventions in real-world settings to improve weight status and healthy growth among low-income children by improving obesity-related behaviors, reducing screen time, and improving sleep. This collection comprises 5 articles exploring factors critical to enhancing stakeholder engagement and implementation fidelity of interventions in racially and ethnically diverse communities (https://www.cdc.gov/pcd/collections/pdf/CORD_Collection.pdf).

PCD has published papers identifying the role that behavioral, psychological, genetic, environmental, biological, and social factors exact on health outcomes among racial and ethnic minority groups. In November, PCD released a fourth collection, focusing on health disparities that was dedicated in honor of Dr Timothy Cunningham, who served as an exceptional PCD associate editor before his untimely death (5). This collection, “Eliminating Health Disparities,” features 9 articles presenting findings on the effectiveness of interventions to reduce the disproportionate burden of chronic diseases among at-risk populations (https://www.cdc.gov/pcd/collections/pdf/Health_Disparities_Collection_2018.pdf).

Today PCD completes the release of its inaugural collection on implementation evaluation, “Promoting the Science and Practice of Implementation Evaluation in Public Health.” It features 5 articles addressing aspects of implementation evaluation conducted in various real-world settings (6). Articles appearing in this collection offer insights into factors that positively or negatively affect the diffusion of proven interventions and the degree of integrity needed to generate success. This inaugural collection offers examples of the kind of research that PCD looks forward to publishing for its Implementation Evaluation article type.

### 2019 Collections

In February 2018, PCD announced a call for papers for its forthcoming collection, “Population Health, Place, and Space: Spatial Perspectives in Chronic Disease Research and Practice.” This collection will focus on articles that help advance the application of spatial statistics, spatial epidemiology, and geographic information systems (GIS) to explore and address spatial variations among many chronic disease-related outcomes, risk factors, and their correlates. PCD received over 90 articles addressing various topics from authors around the world. Careful internal and external review of these 90 articles is under way. We expect to identify an impressive body of articles to be released in mid-2019.

In August 2018, PCD announced a call for papers for its collection “Health Care Systems, Public Health, and Communities: Population Health Improvements.” This collection will focus on research, evaluation, and other work describing innovative and effective ways health care, public health, and communities work together to improve population health. PCD received over 30 submissions. PCD will conduct its usual careful internal and external review of all submissions and anticipates releasing this collection in late 2019.

## Upcoming Additions

PCD is honored that so many authors and readers view the journal as a critical and reliable source of innovative and timely public health research, evaluation, and practice content. In addition to providing our readers with this content, PCD will continue to widely promote articles appearing in the journal. For example, in January 2019, PCD will introduce the use of visual abstracts, an innovative way to display research data, findings, and recommendations. Visual abstracts will serve as a tool to help both the journal and authors gain access to easily digestible content that can be promoted on social media. PCD will also be adding summary boxes to articles. These summary boxes will provide readers a quick snapshot of the article’s content, including information on the public health implications of each article.

## Final Thoughts

PCD thanks authors for their interest in having their work reviewed and published by the journal. PCD will continue to offer authors the opportunity to submit inquiries to the journal to obtain feedback on whether a topic or area of interest is consistent with the journal’s vision and mission. During the past 2 years, PCD received 107 inquiries from authors — all of which received a formal response. Authors interested in submitting inquiries to PCD must provide the journal with all requested information. Authors interested in submitting an inquiry can do so by following guidance provided on the journal’s website (https://www.cdc.gov/pcd/for_authors/submit_inquiry.htm).

On behalf of all PCD staff members and contractors, editorial board members, associate editors, and peer reviewers, we thank you for your unwavering support. We appreciate receiving your comments and suggestions on how to improve the journal’s ability to respond to the needs of its readership. We look forward to an exciting 2019 and working closely with authors to continue our commitment to publish articles of the highest quality.

## References

[1] Jack L Jr . Using *PCD*’s first-ever external review to enhance the journal’s worldwide usefulness to researchers, practitioners, and policy makers. Prev Chronic Dis 2018;15:E41. 10.5888/pcd15.180133 29625629 PMC5894299

[2] Rutledge GE , Lane K , Merlo C , Elmi J . Coordinated approaches to strengthen state and local public health actions to prevent obesity, diabetes, and heart disease and stroke. Prev Chronic Dis 2018;15:E14. 10.5888/pcd15.170493 29369756 PMC5798214

[3] Jack L Jr . Shaping future generations of public health researchers: *Preventing Chronic Disease*’s Student Research Paper Contest. Prev Chronic Dis 2017;14:E96. 10.5888/pcd14.170431 29023229 PMC5645190

[4] Dooyema CA , Belay B , Blanck HM . Implementation of multisetting interventions to address childhood obesity in diverse, lower-income communities: CDC’s Childhood Obesity Research Demonstration Projects. Prev Chronic Dis 2017;14:E140. 10.5888/pcd14.170491 29267154 PMC5743023

[5] Jack L Jr . Advancing health disparities research in population health. Prev Chronic Dis 2018;15:E147. 10.5888/pcd15.180588 30500325 PMC6292139

[6] Jack L Jr . Promoting the science and practice of implementation evaluation in public health. Prev Chronic Dis 2018;15:180645. 10.5888/pcd15.180645 PMC6307834 30576271

